# The Association of Longitudinal and Interpersonal Continuity of Care with Emergency Department Use, Hospitalization, and Mortality among Medicare Beneficiaries

**DOI:** 10.1371/journal.pone.0115088

**Published:** 2014-12-22

**Authors:** Suzanne E. Bentler, Robert O. Morgan, Beth A. Virnig, Fredric D. Wolinsky

**Affiliations:** 1 The Public Policy Center, The University of Iowa, 217 South Quad, Iowa City, IA 52242, United States of America; 2 Division of Management, Policy, and Community Health, The University of Texas School of Public Health, 1200 Herman Pressler, E343, Houston, TX 77030, United States of America; 3 Division of Health Services Research and Policy, University of Minnesota School of Public Health, N504 Boynton, 410 Church St SE, Minneapolis, MN 55455, United States of America; 4 Department of Health Management and Policy, The University of Iowa College of Public Health, N211-CPHB, 105 North River Street, Iowa City, IA 52246, United States of America; Stanford University School of Medicine, United States of America

## Abstract

**Background:**

Continuity of medical care is widely believed to lead to better health outcomes and service utilization patterns for patients. Most continuity studies, however, have only used administrative claims to assess longitudinal continuity with a provider. As a result, little is known about how interpersonal continuity (the patient's experience at the visit) relates to improved health outcomes and service use.

**Methods:**

We linked claims-based longitudinal continuity and survey-based self-reported interpersonal continuity indicators for 1,219 Medicare beneficiaries who completed the National Health and Health Services Use Questionnaire. With these linked data, we prospectively evaluated the effect of both types of continuity of care indicators on emergency department use, hospitalization, and mortality over a five-year period.

**Results:**

Patient-reported continuity was associated with reduced emergency department use, preventable hospitalization, and mortality. Most of the claims-based measures, including those most frequently used to assess continuity, were not associated with reduced utilization or mortality.

**Conclusion:**

Our results indicate that the patient- and claims-based indicators of continuity have very different effects on these important health outcomes, suggesting that reform efforts must include the patient-provider experience when evaluating health care quality.

## Introduction

Continuity of care (CoC) is widely considered to be an essential component of high-quality patient care [1], [2], especially for older adults and those with multiple chronic conditions requiring consistent management and follow-up. Indeed, over a decade ago the IOM declared that CoC was a primary aim in its comprehensive call for national action to transform health care quality [3]. Consequently, new health care delivery models that have CoC as a core element [4], like the patient-centered medical home (PCMH), are an integral component of health care reform today, and are under evaluation by the Center for Medicare & Medicaid Innovation. At present, however, no standard assessment of continuity of care exists. Moreover, there has not been a comprehensive evaluation of the association of continuity of care with subsequent health outcomes and health services use, especially for older adults, although a few studies focusing on single outcomes like mortality and hospitalization for ambulatory care sensitive conditions have recently been reported [5], [6].

Historically, CoC has been difficult to define and quantify, although most definitions consist of at least three components: informational, longitudinal (or provider), and interpersonal (or relational) continuity [7], [8]. Informational continuity results when providers have enough information (typically by maintaining a medical record) about the patient. Having adequate patient information is the first step toward establishing longitudinal continuity which means that the patient has a consistent relationship with a provider in a familiar setting over time. And, conceptually when there is longitudinal continuity, there is a chance for interpersonal continuity to develop. Interpersonal continuity means that knowledge, trust, and respect have developed between the patient and provider over time allowing for better interaction and communication. Within interpersonal continuity, there are both instrumental (provider knowledge about the patient) and affective (mode of provider behavior toward the patient) dimensions that contribute to a good patient-provider relationship [9]. Interpersonal CoC, as reflected in a strong patient-provider relationship, is commonly viewed as the essence of quality primary care [1], [2], [10].

The evidence on the relationship between continuity of care and reduced hospitalization and emergency room visits, and health outcomes has generally suffered from persistent methodologic problems resulting in inconsistent findings that are difficult to [11]–[13]. Most studies examining how CoC relates to patient outcomes have used measures derived from administrative claims as a proxy for provider continuity, under the assumption that repeated contact with a particular provider (longitudinal continuity) equates to having a strong patient-provider relationship (interpersonal continuity) [14], [15]. Recent studies have shown that patient-reported and claims-based measures tap different dimensions of CoC [16], [17]. Yet, few measures reflecting the patient experience of both longitudinal and interpersonal CoC were developed until recently [18]–[20]. Therefore, little is known about how patient CoC experiences relate to patient outcomes and whether those relationships are consistent with those found when claims-based CoC measures were used.

It is unclear whether the distinction between longitudinal continuity and interpersonal continuity matters when it comes to understanding the specific aspects of continuity associated with improved health outcomes and service utilization [7], [8], [15], [17]. The purpose of this study was to examine the association of both patient-reported and claims-based CoC measures with emergency department (ED) use, hospitalization, and mortality among Medicare beneficiaries over a five-year period. We used survey data from 1,219 fee-for-service (FFS) Medicare beneficiaries who participated in the 2004 National Health and Health Services Use Questionnaire (NHHSUQ) [21] linked to their Medicare claims for 2002 through 2009. Using both survey and claims data allowed us to assess two key aspects of continuity (care provided over time and the patient-provider relationship) and to comprehensively evaluate the association of continuity of care with the long-term health and health services use of Medicare beneficiaries.

To our knowledge, we are the first to compare the associations of both patient-reported and claims-based continuity measures with health outcomes in the same study. The findings of this study could have profound implications for policymakers and others involved in deciding on health care delivery system changes designed to promote continuity of care. For example, based on the results of a recent study showing an association between two different claims-based continuity measures and a reduction in preventable hospitalizations [6], a companion commentary suggested primary care practices should regularly measure continuity of care and provide feedback to clinicians, increase the number of days each clinician sees patients, institute same-day or next-day scheduling, and train front desk staff to enforce continuity through visit scheduling [22]. And, while these changes will promote visit-based, longitudinal continuity with a provider (mainly assessed using administrative claims), they may have little effect on improving what happens with the patient during the visit (interpersonal continuity). In fact, some proposed changes could be damaging to the provider-patient relationship, especially if visit length must be shortened to accommodate more flexible scheduling. Thus, if interpersonal continuity (as identified through patient-reports) is found to have a beneficial relationship with the health and service utilization of older adults, then policies that focus solely on promoting longitudinal continuity without considering the impact on the patient-provider experience could be counterproductive to improving health care quality.

## Methods

### Study Design, Data Sources, and Sample

We used information from Medicare beneficiaries who completed the 2004 NHHSUQ merged with their Part A (Institutional) and Part B (non-Institutional) Medicare claims from 2002–2009. It was mailed in the fall of 2004 to a stratified random sample of 6,060 community-residing Medicare beneficiaries 65 years or older. Sampling fractions were varied in order to obtain equal numbers of participants with regard to race/ethnicity (white, black, Hispanic), Medicare plan type (Medicare fee-for-service (FFS) or Medicare managed care (MMC)), sex, and population density [21]. The response rate after adjusting for ineligible survey recipients (e.g., non-community residing, moved out of geographic area, or deceased) was 53% (2,997/5,697). Both the Baylor College of Medicine and University of Iowa institutional review boards approved this study.

The analytic sample was identified as follows. We started with the 2,620 Medicare beneficiaries who had complete data for the 13 continuity-related NHHSUQ items that were used to derive the patient-reported CoC measure. There were only modest differences in a few variables between the 2,620 who completed all items and the 377 who did not [18]. We then linked their survey data to their Medicare claims (Part A and Part B) for 2002–2009, and calculated the claims-based CoC measures using unique Physician Carrier and Outpatient Facility claims for Evaluation and Management (E&M) visits in the years prior to the survey (2002–2004). NHHSUQ respondents in MMC plans were excluded due to the different billing reporting requirements for Part B claims [23]. Thus, the analytic sample included 1,219 people with complete survey responses who had both Part A and Part B coverage and were not enrolled in managed care when they completed the NHHSUQ.

### Outcome Measures

We evaluated the association of CoC with ED use, hospitalization (non-preventable and potentially preventable episodes), and mortality. All outcome measures were derived using the Denominator (Enrollment file), Part A, and Part B Medicare claims for the five-year period (2005–2009) after the 2004 NHHSUQ.

#### Emergency Department Utilization

We used Current Procedural Terminology (CPT) codes to identify ED visits in the Medicare claims [24]. Visits with CPT codes 99281–99285 (E&M in an ED setting) were considered ED visits. These CPT codes account for 80% of all Medicare expenditures for ED services [25]. We evaluated time to first ED visit after completion of the survey. Follow-up time was the number of days from the survey date to the first occurrence of any one of four events: ED visit, managed Medicare entry, death, or December 31, 2009.

#### Hospitalization

Two types of hospitalization events from the Medicare Part A claims were considered within the five-year surveillance period: potentially preventable hospitalizations and any hospitalizations not categorized as preventable. Potentially preventable hospitalizations were defined as any admission for an ambulatory care sensitive condition (ACSC). ACSCs reflect conditions for which continuity of primary care could reduce the hospitalization risk [26] and include diabetes with short-term or long-term complications, uncontrolled diabetes, lower-extremity amputation among patients with diabetes, chronic obstructive pulmonary disease, hypertension, heart failure, dehydration, bacterial pneumonia, urinary tract infection, and angina. ACSCs were defined using the ICD-9-CM codes specified by the Agency for Healthcare Research and Quality's Prevention Quality Indicators [27]. Separate models were run for each of two outcomes: time to first hospitalization that was not preventable, and time to first preventable hospitalization. Follow-up time was the number of days from the survey date to the first occurrence of one of four events: hospitalization (not preventable or preventable, depending on the model), managed Medicare entry, death, or the end of the data period (December 31, 2009).

#### Mortality

Date of death was obtained from the Medicare enrollment files. The main outcome was time to death calculated by subtracting the survey date from the death date.

### Continuity of Care Measures

#### Patient-Reported

We used a multidimensional, 13-item, patient-reported CoC measure derived from patient responses to a subset of the NHHSUQ questions [18]. We considered the overall 13-item scale and each of its four subscales. Two of those subscales tap longitudinal continuity (Care Site and Provider Duration) and two tap interpersonal continuity (Instrumental and Affective). The Care Site subscale has two items and identifies whether the Medicare beneficiary had a usual care site and the type of that site (i.e., doctor's office, Veterans' Affairs Medical Center, emergency room, or other). The Provider Duration subscale has three items and measures the long-term duration of care with their usual site of care and their long-term and short-term (within the past year) duration of care with their regular doctor. The Instrumental subscale has four items which tap the technical care aspects (i.e., thoroughness of examinations, accuracy of diagnoses, explanations of tests and procedures, and knowledge of health) experienced by the patient from visits with the provider. The Affective subscale has four items which tap the “people skills” aspects of the patient-provider interaction (i.e., provider's interest in you, interest in your medical problems, your satisfaction with the provider, and your comfort level with your provider). We normalized each scale item using z-score transformations because they did not have the same response set. Z-scores within each subscale and for the overall scale were summed, giving each item equal weight in the calculation. Results of sensitivity analyses in which we normalized each subscale using z-scores of its sum (giving each subscale equal weight) were comparable.

#### Claims-Based

Thirteen different claims-based CoC measures were used. In previous work [16], these measures were categorized as concentration measures (which identify continuity based on the density of visits to a particular provider), dispersion measures (which identify continuity based on the number of different types of providers seen), and longitudinal measures (which identify continuity based on seeing the same provider at regular intervals over time). The six concentration indices were: Current Provider of Care, Current Provider of Care – discounted [28], Usual Provider of Care [29], Inverse Number of Providers, Herfindahl Index [30], and the Modified, Modified Continuity Index [31], [32]. The five dispersion indices were: Bice-Boxerman CoC [33], Ejlertsson's K Index [34], the Modified Continuity Index [35]–[36], Known Provider [28], and Sequential Continuity [37]. The two longitudinal indices were Wolinsky's CoC [38] and Site Continuity [39]. Further detail on the calculation of each of these measures can be found elsewhere [16].

All CoC indices (patient-reported and claims-based) were calculated so that higher values represented high levels of continuity. The patient-reported measures reflected the respondent's experience in the year prior to the completion of the survey (2003–2004). With the exception of the Wolinsky continuity measure (which, by definition, required two years of claims), all of the claims-based measures were calculated using claims from the same pre-survey period (2003–2004). Where the distributions allowed, we used a set of indicators for each index contrasting the upper and middle tertiles with the lowest tertile (reference). When distributions were more condensed, we compared those with scores above the median to those with scores below the median.

### Covariates

Factors derived from Andersen's behavioral model of health services utilization [40], [41], which categorizes the use of health services as a function of individuals' predisposing (sociodemographic), enabling (socioeconomic), need (health status), and prior health service utilization characteristics, were used to adjust for potential confounding effects on the association of continuity of care with subsequent health outcomes. Measures of predisposing characteristics were obtained from the NHHSUQ and included patient-reported age (≤70, 71 to 76, and >76), sex, race/ethnicity (white, black, and Hispanic), marital status (married or not), and education (high school education or greater vs. less). Indicators for supplemental insurance (in addition to Medicare), population density (living in a metropolitan area), and a median split of income (≤$20K or >$20K) were also taken from the NHHSUQ, and an indicator of dual use of Medicare and Medicaid was taken from the Medicare enrollment files as measures of enabling characteristics.

Indicators of the need for health services included smoking status, health-related quality of life [42], history of selected serious medical conditions (cancer, diabetes, heart failure, myocardial infarction, high blood pressure, cerebrovascular disease, and lung disease), and a comorbidity indicator for having >3 of the following conditions: arthritis, fracture, vision problems, ulcer, arrhythmia, blood disorder, depression, hypothyroidism, valve problems, high cholesterol, back pain, coronary artery disease, hearing problems, peripheral vascular disease, and fluid/electrolyte disorders. The serious condition and comorbidity indicators were obtained from both the NHHSUQ and prior inpatient claims [43], [44], while the other need indicators were taken only from the NHHSUQ. Finally, several covariates were used to adjust for prior health services use. Two measures derived from the Medicare claims were tertiles of the number of physician E&M visits (0–5, 6–16, and 17+ visits) and the occurrence of any hospitalization in the year before the survey. Physician E&M visits were summed for the 2002–2003 period to measure utilization before the time period used for the continuity of care measures. Three measures came from the NHHSUQ, including medication use (0–1, 2–4, and 5+), receipt of a flu shot, and any ED visit reported in the year prior to the survey.

### Statistical Analyses

Multivariable proportional hazard models were used to model time to first ED visit, first hospitalization, and death [45]. Eighteen models were estimated for each of the four outcomes (ED use, preventable and non-preventable hospitalization, and mortality), with each including only one of the individual CoC measures. In addition to the CoC measure, each model included all of the covariates described above to adjust for potential confounding effects on the association between continuity and the particular outcome. All analyses were conducted using SAS software (version 9.2, SAS Institute, Inc., Cary, NC) and were weighted to adjust for the stratified random sampling survey design [21].

## Results

### Respondent Characteristics

Table 1 displays the characteristics of the 1,219 respondents in the analytic sample. Mean age was 74.7 (SD = 7.2), over half were women, a majority were white (83%), married (61%) and half had at least a high school education (52%). Almost 60% had annual incomes over $20,000 and most lived in an urban setting (67%). The vast majority (89%) had insurance supplementing Medicare, but only 11% were dually enrolled in Medicare and Medicaid. The analytic sample was fairly healthy with two-thirds reporting good to excellent health. The most commonly reported condition was hypertension (79%). Almost half (48%) had at least 3 comorbid conditions; less than 10% were smokers. Health service use in the year before the survey was typical with 29% having at least one hospital stay, 27% visiting the ED at least once, and 43% reporting using 2–4 prescription medications. Two-thirds (66%) had reported receiving a flu shot in the year prior to the survey. The average number of physician E&M visits was 17 (SD = 12) or about 1.5 visits per month. The average number of years in FFS Medicare after survey completion was 4.3 (SD = 1.4) out of a maximum of 5.

**Table 1:** Sample Characteristics (N = 1,219 weighted).

Predisposing Characteristics	Percentage
Age 65–70 years	36%
Age 71–76 years	29%
Age ≥77 years	36%
Female	55%
Race/Ethnicity – White	83%
Race/Ethnicity –Black	9%
Race/Ethnicity – Hispanic	8%
Education > High School	52%
Married	61%
**Enabling Characteristics**	
Income over $20,000	59%
Enrolled in Medicare & Medicaid (Dual User)	11%
Has Insurance Supplemental to Medicare	89%
Lives in Urban Setting	67%
**Need Characteristics**	
Self-Reported Good, Very Good, or Excellent Health^a^	66%
Current Smoker	8%
History of Cancer	26%
History of Diabetes	31%
History of Heart Failure	19%
History of Myocardial Infarction	28%
History of Hypertension	79%
History of Cerebrovascular Disease	26%
History of Lung Disease	30%
Comorbidity: >3 (of 15 possible conditions)^b^	48%
**Health Service Use in year prior to the survey**	
Any Hospitalization	29%
Any ED visit	27%
Physician E&M: 0–5 visits	30%
Physician E&M: 6–16 visits	33%
Physician E&M: 17+ visits	37%
Prescription Medications: 0–1	23%
Prescription Medications: 2–4	43%
Prescription Medications: 5 or more	34%
Received a flu shot	66%

a From the SF-8 Health Survey.

b Includes arthritis, non-hip fracture, vision problems, ulcer, arrhythmia, blood disorder, depression, hypothyroid, valve problems, high cholesterol, back pain, coronary artery disease, hearing problems, peripheral vascular disease, or fluid/electrolyte disorders.

### Continuity of Care Measures

The average CoC scores for both the patient-reported and claims-based measures are provided in Table 2. For the patient-reported measures, the mean scores were well into the upper half of the potential range of scores, indicating fairly high levels of patient-reported continuity. There was more variation in the mean values for the claims-based measures (which could range from 0.0 to 1.0), with most average scores between 0.40 and 0.65. Three of the claims-based measures (Inverse Number of Providers, Bice-Boxerman CoC, and Sequential Continuity) had fairly low average scores (0.33, 0.27, and 0.28, respectively).

**Table 2:** Average continuity of care scores (N = 1,219).

Patient-Reported Continuity of Care (Potential Range of Scores)	Mean	Standard Deviation
Care Site (0–5)	4.8	0.8
Provider Duration (0–16)	9.6	4.9
Instrumental (4–19)	14.8	3.0
Affective (4–19)	15.8	2.8
Patient-Reported CoC (8–59)	45.0	8.5
**Claims-Based Continuity of Care (Potential Range of Scores: 0-1)**		
Current Provider of Care	0.41	0.32
Current Provider of Care (discounted)	0.46	0.32
Usual Provider of Care	0.50	0.29
Herfindahl Index	0.41	0.29
Inverse Number of Providers	0.33	0.29
Modified, Modified Continuity Index	0.59	0.30
Ejlertsson's Index K	0.52	0.32
Bice-Boxerman CoC	0.27	0.26
Modified Continuity Index	0.46	0.27
Sequential Continuity	0.28	0.29
Known Provider	0.62	0.49
High Site Continuity	0.89	0.31
Wolinsky Continuity	0.45	0.50

### Emergency Department Use

Nearly two-thirds (63%) had at least one ED visit during follow-up period. Among those who had at least one ED visit, the average number of visits was 3.2 (SD = 3.3) and the average time to the first visit was 1.9 years (SD = 1.4). Table 3 provides the adjusted hazard ratios (AHRs) from the time to first ED visit for those models where the CoC indicator was statistically significant. Those in both the middle and highest tertiles on patient-reported Instrumental CoC (AHR = 0.79; p<.05 and AHR = 0.75; p<.01, respectively), Affective CoC (AHR = 0.77; p<.05; AHR = 0.76; p<.01, respectively), and patient-reported CoC (AHR = 0.77; p<.01; AHR = 0.68; P<.001, respectively) had reduced risks of ED visits compared to those in the lowest tertile of scores. And, those in the middle tertile (compared to lowest tertile) of scores on the Current Provider of Care Index (AHR = 0.78; p<.01), the discounted Current Provider of Care Index (AHR = 0.79; p<.05), and Inverse Number of Providers (AHR = 0.73; p<.01) also had a reduced risk of ED visits. In a sensitivity analysis using only two years of follow-up data, the results were comparable with the exception that the claims-based continuity measures no longer reached statistical significance.

**Table 3:** Six Proportional Hazards Models of Time to First ED visit (n = 1219).

Model^a^	Continuity of Care	Adjusted^b^ Hazard Ratios (95% Confidence Intervals)
1	Patient-Reported Instrumental Continuity^c^	
	Middle Tertile (13–15.5)	0.79* (0.64,0.97)
	Highest Tertile (16–19)	0.75† (0.62,0.91)
2	Patient-Reported Affective Continuity^c^	
	Middle Tertile (15–16)	0.77* (0.63,0.95)
	Highest Tertile (17–19)	0.76† (0.63,0.90)
3	Patient-Reported Continuity^c^	
	Middle Tertile (38–44.5))	0.77† (0.64,0.93)
	Highest Tertile (45–59)	0.68‡ (0.56,0.82)
4	Claims-Based Current Provider of Care (CPC)^c^	
	Middle Tertile (0.20–0.49)	0.78† (0.65,0.94)
	Highest Tertile (≥0.50)	0.87 (0.72,1.05)
5	Claims-Based CPC (discounted)^c^	
	Middle Tertile (0.27–0.58)	0.79* (0.66,0.95)
	Highest Tertile (≥0.59)	0.89 (0.73,1.08)
6	Claims-Based Inverse Number of Providers^c^	
	Middle Tertile (0.16–0.25)	0.73† (0.60,0.89)
	Highest Tertile (≥0.26)	0.84 (0.68,1.02)

^*^p<.05;

† p<.01;

‡ p<.001

a Each model includes the named CoC measure and the covariates.

b Adjusted for all predisposing, enabling, and need characteristics, as well as health service use.

c Reference category is the lowest tertile of scores.

### Hospitalization

Fifty-six percent had at least one non-preventable hospitalization during the five-year prospective period and 19% had at least one preventable (ACSC) hospitalization. Among those who had at least one non-preventable hospitalization, the average time to first non-preventable hospitalization was 2.1 years (SD = 1.5) and the average number of non-preventable admissions was 2.5 (SD = 2.2). Among those who had at least one preventable hospitalization, the average time to first preventable hospitalization was 2.0 years (SD = 1.6) and the average number of preventable hospitalizations was 1.8 (SD = 1.5). Table 4 provides the AHRs from the time to hospitalization for those models where the CoC indicator was statistically significant. None of the patient-reported CoC indicators was significantly associated with non-preventable hospitalization. Those experiencing moderate levels (middle tertile) of discounted Current Provider of Care continuity (AHR = 0.79; p<.05) had a reduced risk of non-preventable hospitalizations compared to those in the lowest tertile, and those with high levels (highest tertile) of continuity on the Modified, Modified continuity index (AHR = 1.25; p<.05) had an increased risk of non-preventable hospital stays compared to those with low levels.

**Table 4:** Six Proportional Hazards Models of Time to Hospitalization (Preventable and Non-Preventable) (n = 1219).

		Adjusted^b^ Hazard Ratios (95% Confidence Interval)	
Model^a^	Continuity of Care	Non-preventable Hospitalization^c^	Preventable Hospitalization^c^
1	Patient-Reported Affective Continuity^d^		
	Middle Tertile (15–16)	0.80 (0.63,1.00)	0.61* (0.41,0.91)
	Highest Tertile (17–19)	0.95 (0.79,1.14)	0.67* (0.49,0.92)
2	Claims-Based Herfindahl Index^d^		
	Middle Tertile (0.26–0.49)	0.87 (0.72,1.04)	1.52† (1.11,2.09)
	Highest Tertile (≥0.50)	0.89 (0.72,1.10)	1.37 (0.93,2.02)
3	Claims-Based Current Provider of Care (discounted)^d^		
	Middle Tertile (0.27–0.58)	0.79* (0.65,0.96)	1.26 (0.90,1.75)
	Highest Tertile (≥0.59)	1.00 (0.82,1.23)	1.21 (0.84,1.75)
4	Claims-Based Modified, Modified Continuity Index^d^		
	Middle Tertile (0.53–0.75)	1.07 (0.87,1.32)	1.50* (1.01,2.21)
	Highest Tertile (≥0.76)	1.25* (1.02,1.54)	1.64† (1.11,2.44)
5	Claims-Based Ejlertsson's Index K^d^		
	Middle Tertile (0.46–0.71)	0.91 (0.73,1.13)	1.48 (0.98,2.24)
	Highest Tertile (≥0.72)	1.09 (0.88,1.35)	1.74† (1.15,2.61)
6	Claims-Based Modified Continuity Index^d^		
	Middle Tertile (0.38–0.63)	1.03 (0.83,1.28)	1.67* (1.10,2.53)
	Highest Tertile (≥0.64)	0.99 (0.78,1.25)	1.16 (0.74,1.80)

* p<.05;

† p<.01.

a Each model includes the named CoC measure and the covariates.

b Adjusted for all predisposing, enabling, and need characteristics, and health service utilization factors.

c A preventable hospitalization is defined as any hospitalization for an ACSC; Hospitalizations that are not for an ACSC are considered not preventable.

d Reference category is the lowest tertile of scores.

Only patient-reported Affective continuity had a protective association with preventable hospitalization for both those in the middle (AHR = 0.61; p<.05) and the highest tertiles (AHR = 0.67; p<.05) compared to those in the lowest tertile. Four claims-based CoC measures indicated that higher levels of continuity increased the risk of a preventable hospitalization. Moderate (compared to low) levels of continuity on the Herfindahl Index (AHR = 1.52; p<.01) and the Modified Continuity Index (AHR = 1.67; p<.05), high (compared to low) levels of continuity on Ejlertsson's Index K (AHR = 1.74; p<.01) and moderate and high levels of continuity (compared to low levels) as indicated by the Modified, Modified Continuity Index (AHR = 1.50; p<.05 and AHR = 1.64; p<.05, respectively) increased the risk of preventable hospitalizations. And, similar to the analyses of ED use, in a sensitivity analysis of preventable hospitalization using only two years of follow-up data, the results with regard to the patient-reported affective continuity were comparable to those with five years of follow-up and the claims-based continuity measures no longer had statistical significance.

### Mortality

Twenty-two percent died during the five-year prospective observation period with the average time to death being 2.8 years (SD = 1.6). Table 5 provides the AHRs from the time to death analyses for those models where the CoC indicator was statistically significant. Only one CoC indicator, patient-reported Duration continuity, had a statistically significant and protective association with time to death (AHR = 0.37, p-value <.001 for the middle tertile and AHR = 0.54, p-value <.01 for the highest tertile, compared to the lowest tertile). Seven claims-based CoC indicators (Usual Provider of Care, Inverse Number of Providers, Modified Modified Continuity Index, Ejlertsson's Index K, Bice-Boxerman CoC, Modified Continuity Index, and Sequential Continuity) and one patient-reported measure (Site continuity) indicated an increased death hazard associated with higher continuity levels.

**Table 5:** Nine Proportional Hazard Models of Time to Death (n = 1,219).

Model^a^	Continuity of Care	Adjusted^b^ Hazard Ratios (95% Confidence Interval)
1	Patient-Reported Care Site Continuity^c^	2.25^†^ (1.33,3.81)
2	Patient-Reported Duration Continuity^d^	
	Middle Tertile (7.5–14.5)	0.37^‡^ (0.24,0.57)
	Highest Tertile (15–16)	0.54^†^ (0.37,0.80)
3	Claims-Based Usual Provider of Care^d^	
	Middle Tertile (0.36–0.59)	1.49* (1.03,2.15)
	Highest Tertile (≥0.60)	2.30^‡^ (1.56,3.38)
4	Claims-Based Inverse Number of Providers^d^	
	Middle Tertile (0.16–0.25)	1.59* (1.03,2.46)
	Highest Tertile (≥0.26)	1.80* (1.12,2.88)
5	Claims-Based Modified, Modified Continuity Index^d^	
	Middle Tertile (0.53–0.75)	1.32 (0.86,2.03)
	Highest Tertile (≥0.76)	1.69* (1.13,2.52)
6	Claims-Based Ejlertsson's Index K^d^	
	Middle Tertile (0.46–0.71)	1.00 (0.64,1.56)
	Highest Tertile (≥0.72)	1.70* (1.12,2.59)
7	Claims-Based Bice-Boxerman CoC^d^	
	Middle Tertile (0.13–0.31)	1.05 (0.68,1.64)
	Highest Tertile (≥0.32)	2.33^‡^ (1.56,3.49)
8	Claims-Based Modified Continuity Index^d^	
	Middle Tertile (0.38–0.63)	1.42 (0.91,2.22)
	Highest Tertile (≥0.64)	1.98^†^ (1.23,3.21)
9	Claims-Based Sequential Continuity^d^	
	Middle Tertile (0.10–0.33)	2.00^‡^ (1.36,2.96)
	Highest Tertile (≥0.34)	2.35^‡^ (1.59,3.49)

*p<.05; † p<.01; ‡ p<.001.

a Each model includes the named CoC measure and the covariates.

b Adjusted for Medicare managed care entry during the follow-up period, all predisposing, enabling, and need characteristics, as well as health service use factors.

c Reference category is low continuity defined as less than the average score.

d Reference category is the lowest tertile of scores.

Because of the large number of CoC measures (18) evaluated for each of the four outcomes (ED use, non-preventable and preventable hospitalization, and mortality), Table 6 provides a summary of the results for each of the 72 models. With the exception of Care Site, each of the patient-reported continuity measures decreased the hazard of at least one of the outcomes while only three (of a possible 13) of the claims-based continuity measures had a similar effect. In contrast, eight of the claims-based measures increased the hazard of at least one of the outcomes.

**Table 6:** Summary of Results for Emergency Department (ED) Use, Hospitalization, and Mortality for each of the Patient-Reported and Claims-Based Continuity of Care Indicators.

Continuity Indicator	ED use	Non-preventable Hospitalization	Preventable Hospitalization	Mortality
**Patient-Reported Continuity**				
Care Site				+
Duration				−
Instrumental	−			
Affective	−		−	
Full-Scale Continuity	−			
**Claims-Based Continuity**				
Herfindahl Index			+	
Usual Provider of Care				+
Current Provider of Care	−			
Current Provider of Care (discounted)	−	−		
Inverse Number of Providers	−			+
Modified, Modified Continuity Index		+	+	+
Ejlertsson's Index K			+	+
Bice-Boxerman Continuity				+
Modified Continuity Index			+	+
Sequential Continuity				+
Known Provider Continuity				
Wolinsky Continuity				
Site Continuity				

## Discussion

Continuity of care is considered a hallmark of high-performing primary care. There are many different ways to assess whether CoC is provided, but there is little if any consensus about best practices for CoC assessment. Nonetheless, CoC is a fundamental component of current health reform, including the PCMH and Accountable Care Organizations. The reason is that CoC has been associated with preventive and chronic care service use, patient and provider satisfaction, decreasing hospitalization and emergency department (ED) use, lower overall health care expenditures, and lower mortality in older adults [5], [12], [46], [47]. In this study, we examined the relationship of two distinct aspects of CoC (longitudinal and interpersonal) using both patient- and claims-based assessments with three health outcomes important in the care of older adults: ED use, hospitalization, and mortality.

Older adults are the most frequent users of EDs, and often do so for conditions that are non-urgent [48]. Theoretically, high CoC should have its largest effect on reducing ED visits because the established patient-provider relationship enables less severe health issues to be treated outside of the ED setting, reserving ED use for truly emergent situations. Our results indicate that for some claims-based and patient-based CoC indicators, there is a reduction in the risk of ED use associated with higher levels of CoC. Of particular note, moderate and high levels of interpersonal CoC (Instrumental and Affective patient-reported CoC), which are indicative of the patient perspective of the quality of the provider continuity, were most effective at reducing the risk of ED visits, providing evidence to support the value of a good patient-provider relationship [12], [13]. In fact, when we looked at the number of patients who would need to move from the lowest level of affective (or instrumental) continuity to at least the moderate (or middle tertile) level of affective (or instrumental) continuity in order to avoid one emergency department visit within a two year period [49], we found that 14 people would need to experience higher continuity in order to obtain such a reduction in emergency department visits. Thus, health plans could give incentives to providers to improve the quality of their continuous care relationships with their patients and the payout in the form of reduced emergency department visits could be substantial.

Reducing hospitalizations, particularly those that are potentially preventable with adequate outpatient care (ACSCs), is both a care-quality and expenditure-containment goal [50], [51]. High CoC should be an effective tool for meeting this goal. In this analysis, however, the results were mixed. For preventable hospitalizations, high levels of CoC on several claims-based measures increased the risk of hospitalization. This may reflect a confounding effect due to unmeasured comorbidity and disease-severity. Or, it may reflect the fact that as individuals age, it is simply difficult for CoC to have much of an effect on hospitalization. Patients are hospitalized when they are too sick to be cared for at home and the likelihood of this occurring increases dramatically with age. Another possible explanation is that patients who are non-compliant with treatment regimens become sicker which results in more visits to their physician in conjunction with a higher likelihood of hospitalization.

Thus, further research on the effects of CoC on hospitalization (both non-preventable and preventable) is needed. This is especially important given a recent report of a modest 2% reduction in the risk for preventable (ACSC) hospitalizations associated with moving from no continuity on two claims-based CoC measures to complete continuity [6]. In sensitivity analyses, we were unable to replicate those results using the same two CoC measures (Bice-Boxerman CoC and Usual Provider of Care). However, as with ED use, high levels of affective interpersonal continuity had a protective association with ACSC hospitalizations. It may be that establishing a caring, trusting bond as part of the patient-provider relationship helps both the patient and provider understand when outpatient and home care can substitute for hospitalization. This is a particularly salient finding because ACSC hospitalizations are potentially preventable with quality care in the outpatient setting, and interpersonal continuity is viewed as foundational to the provision of quality care.

Only one CoC measure, patient-reported Duration continuity, had a significant protective association with mortality for Medicare beneficiaries. In contrast, for eight other CoC measures, higher CoC increased the likelihood of death. A previous study reported a protective association of CoC with mortality among older adults [5], but found that the magnitude of the association diminished with increased cumulative exposure to CoC. Like that study, our analysis may suffer from confounding due to unmeasured comorbidity and condition severity. Or, it may simply be that our results reflect the likelihood that as older patients become more seriously ill, they tend to see their physicians more regularly, resulting in higher CoC levels. Given the severity of their illnesses, however, even high CoC levels may not be sufficient to alter their life course. Thus, higher CoC levels may reflect the combined illnesses that eventually lead to their death rather than the quality of their continuity of care. Alternatively, a comfortable and satisfactory relationship with a provider may encourage higher CoC levels in healthier patients because they are less inhibited about scheduling a visit even for minor medical events [52], [53].

Our study is not without limitations. One is that we were not able to calculate every extant claims-based CoC measure. We were, however, able to recreate the two measures most commonly used in assessments of health outcomes (i.e. Usual Provider of Care and Bice-Boxerman CoC). Another limitation is that the cross-sectional design of the survey made it impossible to use time-dependent patient-reported CoC measures in our analyses, thereby limiting our ability to adequately tease out how adverse health may affect continuity over time. Continuity of care may have a limited role in preventing ED visits that occur when a patient's regular provider's office is not open. Our data did not allow us to tell if an ED visit occurred after regular business hours so we were not able to evaluate this potentiality. Yet another limitation is that we were only able to use the survey data and claims from respondents in FFS Medicare due to differential reporting requirements in managed care Medicare plans. Because choice of providers may be limited within some (especially closed panel) MMC plans, these findings may not generalize to beneficiaries in Medicare Advantage plans. Finally, these findings relate to the experiences of older Medicare beneficiaries and may not generalize to younger people.

Continuity of care has long been advocated as an integral part of the delivery of quality primary care and several studies have evaluated its effect on outcomes with mixed results. In part, this heterogeneity stems from the measures used for assessing continuity. Most studies have used administrative claims to tap provider continuity through visit-based utilization. Few studies have included patient experiences of continuity when assessing its relationship to health outcomes, despite the increased advocacy for including the patient experience. The results of our study arm policymakers and health system evaluators with the knowledge that the patient experience of continuity and continuity as assessed through billing claims have different effects on several important health and health service utilization outcomes for Medicare beneficiaries. In particular, the association of high interpersonal continuity (which cannot be assessed from billing claims) with reduced risk of ED use and preventable hospitalization suggests that health care reform components embodied in the PCMH to enhance a strong patient-provider relationship over time should be promoted for older adults.
